# Comprehensive immune profiling of patients with advanced urothelial or renal cell carcinoma receiving immune checkpoint blockade

**DOI:** 10.3389/fonc.2022.973402

**Published:** 2022-09-13

**Authors:** Jean-Michel Lavoie, Priya Baichoo, Elizabeth Chavez, Lucia Nappi, Daniel Khalaf, Christian K. Kollmannsberger, Kim N. Chi, Andrew Weng, Christian Steidl, Bernhard J. Eigl, Michael Nissen

**Affiliations:** ^1^ Department of Medical Oncology, BC Cancer – Surrey Centre, Surrey, BC, Canada; ^2^ Terry Fox Laboratories, Vancouver, BC, Canada; ^3^ Centre for Lymphoid Cancer, BC Cancer Research Centre, Vancouver, BC, Canada; ^4^ Department of Medical Oncology, BC Cancer – Vancouver Centre, Vancouver, BC, Canada; ^5^ Vancouver Prostate Centre, Department of Urologic Sciences, University of British Columbia, Vancouver, BC, Canada

**Keywords:** checkpoint blockade, immunotherapy, clinical outcomes, cytometry, scRNAseq

## Abstract

Immune checkpoint inhibitors (ICI) are used in the treatment of urothelial and renal cell cancers. While some patients may have exceptional responses, better predictive biomarkers are needed. We profiled the circulating immune compartment of patients receiving ICI to identify possible immune markers associated with immunotherapy response or resistance. Peripheral blood samples were collected prior to, and 3 weeks after initiation of ICI. Using mass cytometry, 26 distinct immune populations were identified. Responders to immune checkpoint inhibitors had higher frequencies of naïve CD4+ T-cells, and lower frequencies of CD161+ Th17 cells and CCR4+ Th2 cells. Non-responders had a higher frequency of circulating PD1+ T-cells at baseline; there was a subsequent decrease in frequency with exposure to ICI with a concomitant increase in Ki67 expression. Flow cytometry for cytokines and chemokine receptors showed that CD4+ T cells of non-responder patients expressed less CXCR4 and CCR7. In addition, their PD1- CD4+ T cells had higher TNFα and higher CCR4 expression, while their PD1+ CD4+ T cells had higher interferon γ and lower CCR4 expression. The role of γ/δ T-cells was also explored. In responders, these cells had higher levels of interferon γ, TNFα and CCR5. One patient with a complete response had markedly higher frequency of γ/δ T-cells at baseline, and an expansion of these cells after treatment. This case was analyzed using single-cell gene expression profiling. The bulk of the γ/δ T cells consisted of a single clone of Vγ9/Vδ2 cells both before and after expansion, although the expansion was polyclonal. Gene expression analysis showed that exposure to an ICI led to a more activated phenotype of the γ/δ T cells. In this study, the circulating immune compartment was shown to have potential for biomarker discovery. Its dynamic changes during treatment may be used to assess response before radiographic changes are apparent, and these changes may help us delineate mechanisms that underpin both response and resistance to ICI. It also hypothesizes a potential role for γ/δ T cells as effector cells in some cases.

## Introduction

Blockade of the Programmed Death 1 (PD-1)/PD-1 Ligand (PD-L1) immune checkpoint axis with monoclonal antibodies is now a widespread therapeutic strategy across many cancers, including renal cell (1) and urothelial carcinomas (2) (RCC and UC). While large clinical trials have established that this approach is superior to previous standard treatments, many patients do not respond or have short lived response and, especially in UC the benefit is largely driven by a small fraction of patients with durable responses. Currently, there is no validated predictive biomarker to help identify which patients will respond to PD-1/PD-L1 blockade in RCC and UC, although many biomarkers have been explored.

In other cancers where PD-1/PD-L1 blockade is also a standard of care, specific circulating immune cell populations have been associated with response. For instance, in patients with advanced melanoma, the degree of classical monocytes present prior to treatment with the PD-1 inhibitor pembrolizumab has been correlated with response and survival (3). A different cohort of patients with melanoma treated with pembrolizumab has shown an association between re-invigoration of exhausted immune T-cells after exposure and clinical response (4). This contributes to the mounting body of evidence that the circulating immune compartment plays a key role in the response to checkpoint blockade. Recently, Yost et al. demonstrated that PD-1 blockade led to a replacement of immune-infiltrating T-cell clones in patients with basal or squamous cell skin cancer (5). Conversely, in urothelial carcinoma, peripheral expansion of tumor-infiltrating T-cell clones was observed in patients with clinical benefit from the PD-L1 inhibitor atezolizumab (6). Other indirect measures such as the pattern of response observed in patients with non-small cell lung cancer and microsatellite unstable gastrointestinal cancers support the hypothesis that systemic immunity is a key component of the response to checkpoint inhibition (7). Therefore, circulating immune cells are a rich target for biomarker discovery. Although they cannot directly demonstrate phenomena in the tumour microenvironment, changes in systemic immunity may still provide insight into the underlying biology of response to immune checkpoint inhibition.

Our study aimed to characterize the circulating immune compartment of patients with advanced UC and RCC receiving PD-1 blockade. By tracking changes in a broad array of populations, we demonstrate reinvigoration of immune-exhausted T-cells, as well as potential trafficking of these cells through chemokine signalling. We also identified a case of γδ-T-cell-mediated exceptional response to checkpoint inhibition. This subset of T-cells has recently been identified as a potential mechanism of response to checkpoint inhibition, and we therefore aimed to characterize this γδ-T-cell population through single-cell RNA sequencing.

## Materials and methods

### Sample acquisition and processing

PD-1/PD-L1 inhibitors naïve adult patients with advanced RCC or UC enrolled in the British Columbia provincial genito urinary biobank research project were eligible for this study. Whole blood samples were collected prior to initiation of therapy (baseline), and 3 weeks after the first dose of anti-PD1/PD-L1 (+/- 5 days, follow-up). These timepoints were selected to assess whether changes in circulating immune cells can be identified before radiographic changes would be expected. Moreover, previous reports had shown that in melanoma changes could be found as early as 3 weeks after initiating treatment (4).

Red blood cells were lysed using an ammonium chloride buffer and remaining preipheral blood monomuclear cells (PBMCs) were cryopreserved using liquid nitrogen in 90% FBS + 10% DMSO freezing media for batched analysis. All study procedures were conducted in agreement with the Declaration of Helsinki and were approved by the BC Cancer Research Ethics Board. Response to treatment was assessed by the investigators using the RECIST criteria version 1.1 (8).

### Mass cytometry

Cryopreserved PBMCs were thawed to 37 degrees and 5x10^6^ viable cells per sample were taken for staining. Non-viable cells were stained with 113Cd-tagged maleimide, and Fc receptors were blocked using TruStain Human FcX (BioLegend) to inhibit nonspecific staining. Cells were incubated in a panel of metal-tagged antibodies (**Table S1**) targeted against surface antigens before fixation and permeabilization using the FoxP3 Transcription Factor Staining Kit (ThermoFisher) as per manufacturer’s instructions, followed by staining with a panel of metal-tagged antibodies directed against intracellular antigens. Cells were then barcoded using the 20-Plex Pd Barcoding Kit (Fluidigm) as per manufacturer’s instructions and pooled together. Nuclei were then stained with Cell-ID Iridium DNA Intercalator overnight before acquisition on a CyTOF2 instrument (Fluidigm). Signal normalization was performed using EQ Four-Element Calibration Beads (Fluidigm), and samples were normalized and debarcoded after acquisition using published tools as outlined in (9).

### Flow cytometry

PBMCs were thawed as described above. For measurement of chemokine receptor expression, cells were stained immediately after thawing with fluorophore-tagged antibodies directed against chemokine receptors for 15 minutes at 37 degrees, then fluorophore-tagged antibodies directed against other cell surface molecules were added and cells were further stained for 15 minutes at 4 degrees. After antibody staining, non-viable cells were labelled with Live/Dead Fixable dye (ThermoFisher) and acquired using a FACS Symphony A5 instrument (BD). For measurement of cytokine production, once PBMCs were thawed they were allowed to rest for 2 hours to recover from cryopreservation before 4 hours of stimulation with 1µg/mL ionomycin, 10ng/mL PMA, 1µg/mL brefeldin A and 2µM monensin. Non-viable cells were labelled with Live/Dead Fixable dye and incubated with a panel of fluorophore-tagged antibodies directed against surface markers. Cells were then fixed and permeabilized using the CytoFix/CytoPerm kit (BD) according to manufacturer’s instructions and stained with a panel of fluorophore-tagged antibodies directed against intracellular cytokines before being acquired using a FACS Symphony A5 instrument.

### Single-cell RNAseq

PBMCs were thawed as described above and suspended in a solution of 100ng/mL DAPI in PBS + 2% FCS for FACS sorting. Total viable cells were sorted using a BD Melody instrument for further processing. In total, 8700 cells per sample were loaded into a Chromium Next GEM Chip G and processed according to the Chromium Next GEM Single Cell V(D)J Reagent Kits V1.1 User Guide. Expression libraries were constructed using the Chromium Next Gem Single Cell 5’ Library and Gel Bead Kit v1.1 and Chromium i7 Multiplex Kit. TCRγ/δ transcripts were amplified by using the 2 step PCR protocol as described by Mimitou et al. (10). Enriched TCR libraries were constructed according to the Chromium Next GEM Single Cell V(D)J Reagent Kits V1.1 User Guide. Gene expression and TCR libraries were pooled at a ratio of 6:1 and sequenced in a NextSeq 550 instrument (High Output Flow Cell V2.5 Recipe: 27 cycles Read1, 8 cycles index, 125 cycles Read2). CellRanger software (v3.1.0; 10X Genomics) was used to demultiplex the raw data, generate quality metrics, and generate per-gene count data for each cell.

### Data analysis

CyTOF data were analysed in FlowJo to manually exclude debris, dead cells, and doublet events, before import into R for downstream analysis. Expression data were transformed using standard hyperbolic arc-sine (transformation coefficients a=0, b=0.2) and populations were defined using the Phenograph clustering algorithm. Each population was then annotated by manual inspection of canonical surface markers before interpretation. Flow cytometry data were analysed manually in FlowJo. scRNAseq data were analysed in R using the Monocle pipeline (version 2 (11),). Statistical analyses were carried out in R, using non-parametric ANOVA models to determine the effects of clinical response and sampling time on the abundance and phenotype of immune populations. Statistical significance was defined at a threshold of p<0.05 for all tests.

## Results

### Frequencies of specific immune populations correspond with clinical response to checkpoint blockade

Ten patients were enrolled; nine were able to provide pre- and post-treatment samples; one patient progressed rapidly and was unable to provide a follow-up sample. None of the patients had active infections or recent antibiotic use. Patient’s primary histology, type of treatment and response to treatment are shown in **Table 1**. Among all 19 samples, 26 immune cell populations were identified by Phenograph clustering. Major populations were present in all samples, while some rare populations were absent from some cases.

**Table 1:** Baseline characteristics and response to checkpoint inhibition.

Case ID	Primary malignancy	Histology/grade	Treatment	Response
1	Urothelial	High-grade	Pembrolizumab	PD
2	Urothelial	High grade	Durvalumab plus cisplatin/gemcitabine	PR
3	Renal cell	Clear cell, grade 3	Nivolumab/ipilimumab	PR
4	Renal cell	Clear cell, grade 3	Nivolumab/ipilimumab	PD
5	Renal cell	Clear cell, grade 3	Nivolumab/ipilimumab	SD
6	Urothelial	High-grade	Durvalumab plus cisplatin/gemcitabine	CR
7	Urothelial	High grade	Pembrolizumab	PD
8	Urothelial	High-grade	Pembrolizumab	SD
9	Urothelial	High-grade	Pembrolizumab	PR
10	Renal cell	Clear cell, grade 3	Nivolumab	PD

**Figure 1:**
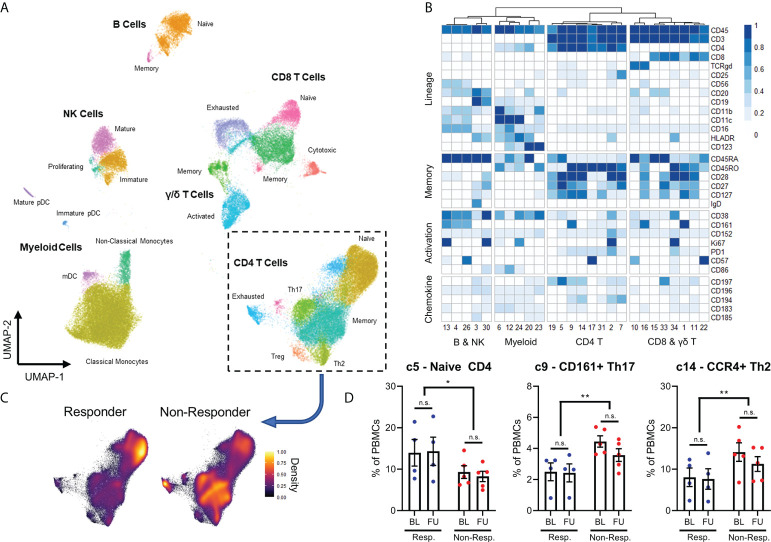
Expression patterns of 26 immune populations found in 19 samples represented through dimensional reduction **(A)** and heatmap **(B)**. Differences in density of different CD4+ T-cells populations between responders and non-responders are observed visually **(C)**, with responders having significantly higher proportion of naïve CD4 T-cells, lower CD161+ Th17 T-cells and CCR4+ Th2 cells seen both at baseline (BL) and at a 3-week follow-up (FU) **(D)**. N.S.: not significant; *p<0.05; **p<0.01.

While markers for granulocytes were included in the mass cytometry panel (shown in **Supplementary Table 1**), these cells were excluded from analysis due to low viability and the presence of artifacts, likely owing to the cryopreservation process. The populations are represented using dimensional reduction (UMAP) in **Figure 1A**, and expression profiles of these populations are represented by heatmap in **Figure 1B**.

Interestingly, all 26 populations did not display significant changes in frequency between baseline and follow-up samples, remaining stable over the course of the immunotherapy. When we compared the immune compositions of responder patients against non-responder patients by ANOVA, we discovered that responders had higher frequencies of naive CD4+ T cells (cluster 5) and lower frequencies of CD161+ Th17 cells (cluster 9) and CCR4+ Th2 cells (cluster 14). These changes are shown by a change in density across CD4 T cell populations in **Figure 1C**, and quantified in **Figure 1D**. This effect was observed prior to PD-1 checkpoint blockade, and like other populations, remained stable until follow-up sampling three weeks later.

All other populations were not significantly different between responder and non-responder patients. In addition, there was no difference between patients with urothelial and renal cell cancer, and whether patients received single-agent PD-1 blockade, or in combination with chemotherapy or CTLA-4 blockade (results not shown). These results demonstrate an overall more activated phenotype amongst the CD4+ T cells of cancer patients who fail to respond to checkpoint inhibitor therapy. This is observed irrespective of the specific checkpoint inhibitor used or the primary histology.

### Checkpoint blockade alters the frequency and characteristics of PD1+ T cells in non-responder patients

**Figure 2:**
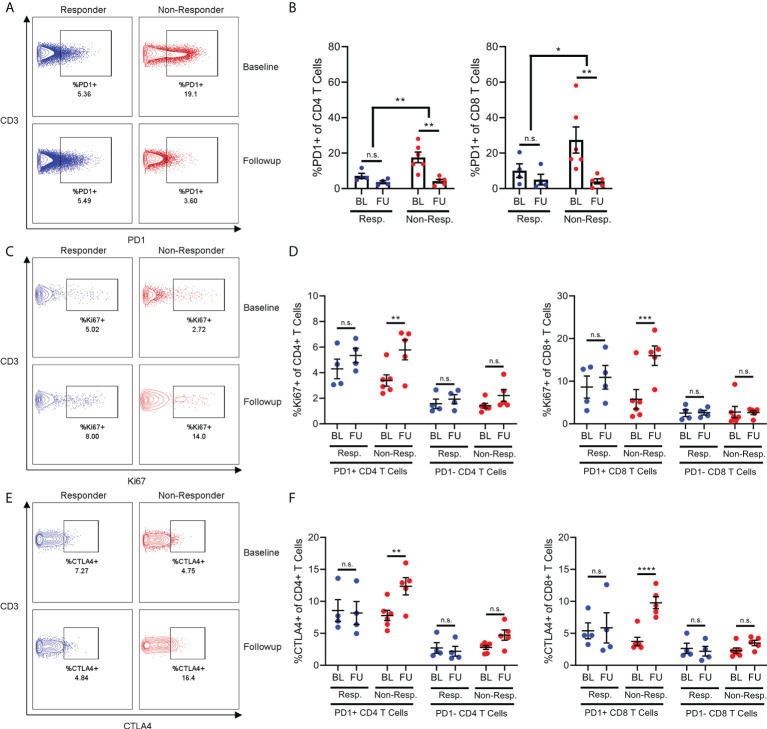
The frequency of PD1+ T-cells in non-responders decreases following exposure to checkpoint inhibition **(A, B)**, but this is associated with a significant increase in expression Ki67 **(C, D)** and CTLA4 expression **(E, F)** in these cells. N.S, not significant; *p<0.05; **p<0.01; ***p<0.001; ****p<0.0001.

In addition to quantifying immune cell populations, we studied the expression of functional and exhaustion markers by immune cells, with a particular focus on T cells. We discovered that non-responder patients had higher frequencies of PD1+ cells among both CD4+ and CD8+ T cell compartments compared to responder patients, but only in baseline samples (**Figures 2A, B**).

Interestingly, the frequency of PD1+ T cells were reduced after immunotherapy in non-responder patients, which then resembled responder patients in follow-up samples. Given this interesting dynamic, we then quantified expression of both the proliferation marker Ki67 and the inhibitory marker CTLA-4 in order to gain an appreciation of how the behaviour of these PD1+ T cells might be changing after immunotherapy. Surprisingly, the expression of Ki67 increased in the PD1+ T cells of non-responder patients between baseline and follow-up, despite the overall frequency of PD1+ T cells decreasing (**Figures 2C, D**). This effect was not observed in the PD1+ T cells from responder patients, nor was it observed in the PD1- T cells of either responder or non-responder patients. The same effect was also observed regarding the expression of CTLA-4 (**Figures 2E, F**).

These results both suggested that PD1+ T cells from non-responder patients were receiving stimulation during immunotherapy, as T cells both proliferate and begin expressing inhibitor receptors after strong stimulation. However, this evidence of proliferation and activation of PD1+ T cells was contradicted by the decrease in frequency. Therefore, we hypothesized that these PD1+ T cells were indeed becoming activated, but then migrating from the blood and into lymphoid organs or inflamed tissues such as the tumor site. Specifically, distinct differences in chemokine profiles between the T cells of responder and non-responder patients suggests migration into inflamed tissue or secondary lymphoid organs plays a role in differential frequencies of T cell populations.

### Functional characteristics of PD1+ and PD1- T cells are distinct in responder patients

In order to further explore our hypothesis of differential activation and migration in PD1+ T cells from non-responders, we used flow cytometry to quantify the expression of a range of chemokine receptors and cytokines on PD1+ and PD1- T cells from responder and non-responder patients. As with immune populations, the differences in chemokine receptor and cytokine expression between responder and non-responder patients centered on CD4+ T cells.

**Figure 3:**
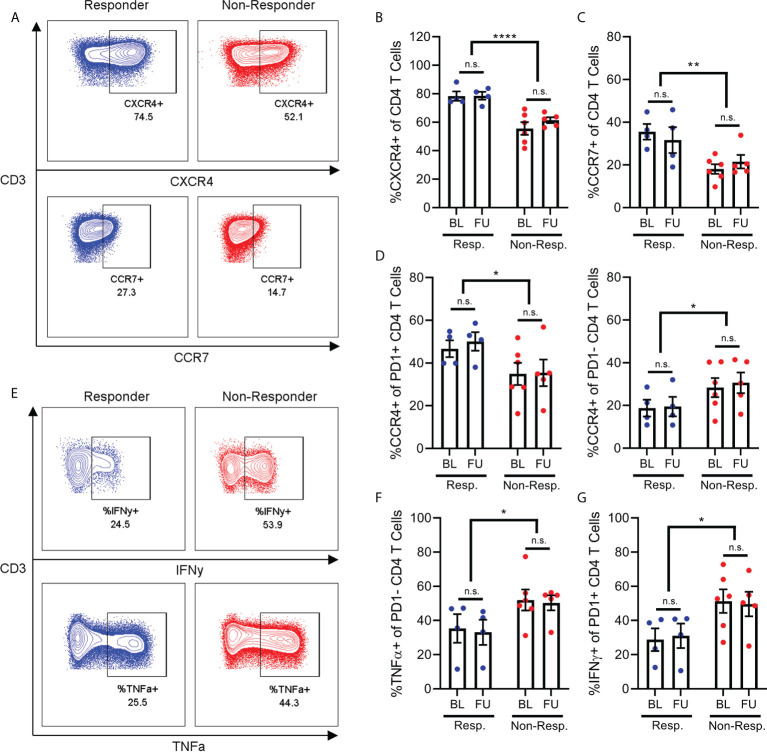
Chemokine receptor and cytokine expression on CD4+ T-cells. Frequency of CD4+ T cells **(A)** expressing CXCR4 **(B)** or CCR7 **(C)** are reduced in non-responder patients compared to responders. Frequency of PD1+ T cells expressing CCR4 is decreased in non-responder patients, while frequency of PD1- T cells expressing CCR4 is increased in non-responders **(D)**. Frequency of PD1- CD4 T cells (**E**, top) expressing IFNy **(F)**, or frequency of PD1- CD4 T cells (E, bottom) expressing TNFa **(G)** are both increased in non-responder patients. N.S, not significant; *p<0.05; **p<0.01 ****p<0.0001.

Specifically, the CD4+ T cells of non-responder patients expressed less CXCR4 and CCR7 compared to the CD4+ T cells of responder patients (**Figures 3C, D**). This aligns with previous findings that non-responder patients have lower frequencies of naive CD4+ T cells, since CXCR4 and CCR7 are expressed on these cells (4). Furthermore, we found that CD4+ T cells from non-responder patients expressed increased amounts of the cytokines TNFα and IFNy relative to responder patients. Interestingly, the increased expression of TNFα was specific to PD1- CD4+ T cells (**Figure 3F**), while the increase in expression of IFNy was specific to PD1+ CD4+ T cells (**Figure 3G**).

Finally, we found that the effect of treatment response on CCR4 expression by CD4+ T cells was dependent on the PD1 status of those cells. Specifically, non-responder patients express less CCR4 on their PD1+ CD4+ T cells relative to responder patients (**Figure 3D**), while conversely, non-responder patients also express significantly more CCR4 on their PD1- CD4+ T cells (**Figure 3D**). We observed a minor increase in expression of CXCR3 in PD1+ T cells in follow-up samples compared to baseline, however this was observed in both responder and non-responder patients. Other chemokine receptors studied but that were not differentially expressed include CCR5, CCR6 and CXCR5 (data not shown).

These results reinforce our finding that the PD1+ CD4+ T cells of non-responder patients are in a more activated state. However, the reduced expression of chemokine receptors on these PD1+ cells does not explain the lower frequency of these cells in the PBMCs of non-responder patients. As such, this depletion of PD1+ T cells from the bloodstream of non-responder patients may be driven by chemokine receptors other than those included here, or other T cell-extrinsic factors.

### A role for gamma/delta T cells in treatment response

**Figure 4:**
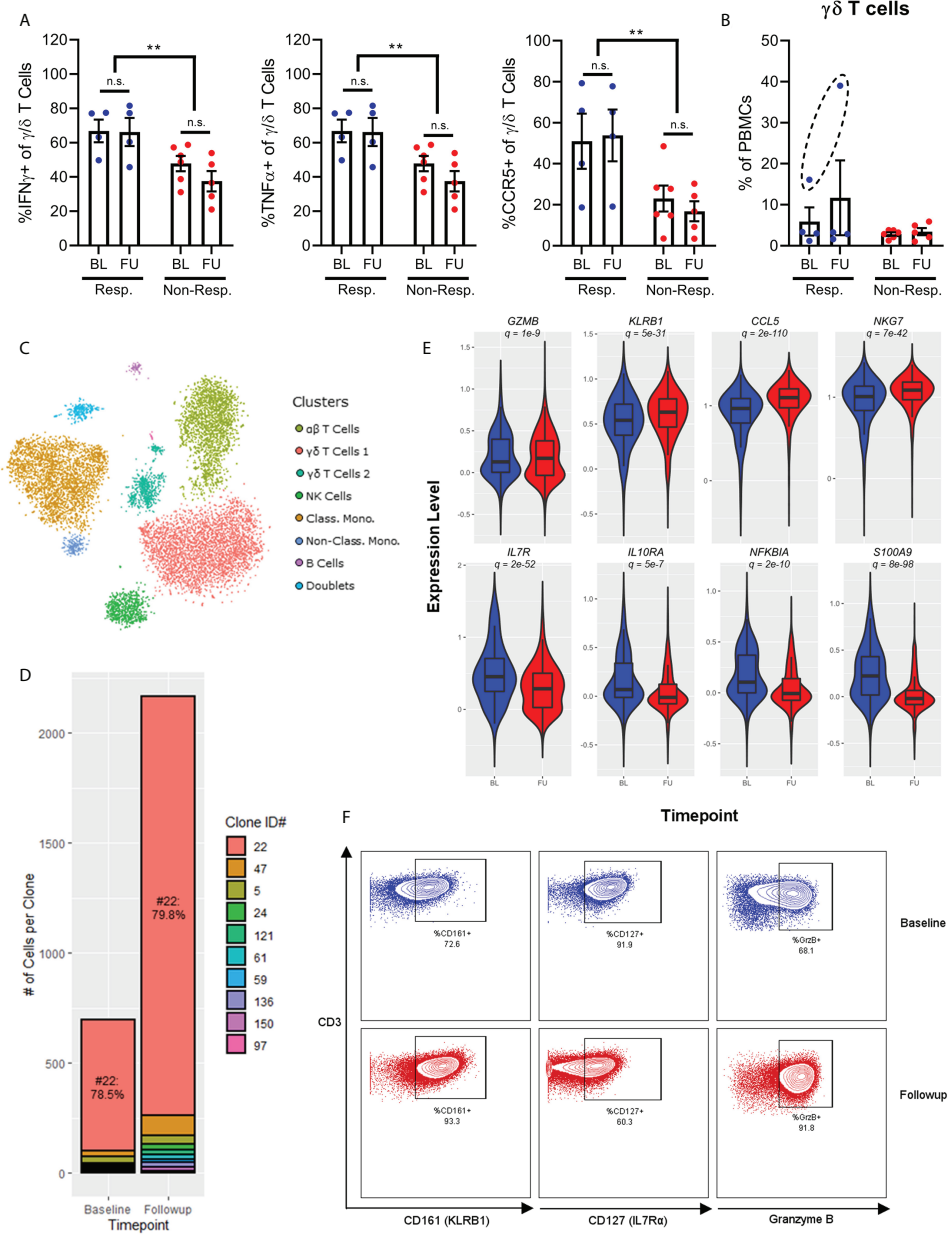
Chemokine receptor and cytokine expression on γ/δ T-cells show increased expression of IFNγ, TNFa and CCR5 in responders **(A)**. Frequency of γ/δ T-cells before and after treatment shows one patient with significantly higher expression at baseline, and expansion after treatment **(B)**. Single-cell RNAseq of a patient with complete response to checkpoint inhibition shows 7 broad populations **(C)**. There is a polyclonal expansion of γ/δ T-cell dominated by a Vγ9/Vδ2 clone **(D)**. A violin plot of single-cell RNAseq of the γ/δ T-cells shows an increased activated phenotype after exposure to checkpoint inhibitor (**E**, baseline in blue and post-treatment in red), which is confirmed by flow cytometry **(F)**. NS, not significant; **p<0.01.

While our results thus far have focussed on the roles of conventional CD4+ and CD8+ α/β T cells in treatment response, our findings also suggest a role for the unconventional γ/δ subset of T cells. Specifically, the γ/δ T cells of responder patients produced significantly higher amounts of both IFNy and TNFα (**Figure 4A**), two cytokines which were conversely associated with non-responder patients when produced by conventional α/β T cells (**Figure 3**). Furthermore, γ/δ T cells from responder patients expressed significantly higher levels of the chemokine receptor CCR5 (**Figure 4A**). Together, these results suggest that activated γ/δ T cells migrating into inflamed tissue are associated with good treatment response, which is in stark contrast to our prior findings that an activated state in conventional α/β T cells with reduced expression of chemokine receptors is associated with poor outcomes.

γ/δ T cells typically represent 1-5% of PBMCs. However, one patient in our cohort, who was the only patient to benefit from a complete response to anti-PD1 immunotherapy, displayed a much higher frequency of these cells. This patient received durvalumab in combination with cisplatin and gemcitabine for urothelial cancer. In this patient, γ/δ T cells represented 15% of total PBMCs at baseline, and then expanded to 40% at follow-up (**Figure 4B**). This observation was unique among the cohort for two reasons. First, γ/δ T cells only represented ~3% of PBMCs from other patients regardless of sampling time, and second, no other immune cell population was significantly changed between time points. Given the seemingly unique nature of these cells, we performed single-cell RNA and TCR sequencing on PBMCs from this patient in order to gain a deeper understanding of both the gene expression profile and clonal dynamics of these cells.

Single cell gene expression profiling of PBMCs discriminated 7 broad populations based on tSNE dimensionality reduction followed by Leiden clustering (α/β T cells, 2x γ/δ T cells, NK cells, classical monocytes, non-classical monocytes, and B cells) and one cluster of doublet events, which were excluded from further analysis (**Figure 4C**). Interestingly, single-cell TCR analysis determined that the bulk of the γ/δ T cells (79%) consisted of a single clone of Vγ9/Vδ2 cells (denoted #22) both at baseline and after follow-up sampling (**Figure 4D**). Despite this clonal dominance, the expansion of γ/δ T cells was distinctly polyclonal, with multiple smaller clones expanding after immunotherapy to approximately the same degree as the dominant clone. Furthermore, investigation of the gene expression profiles of these cells annotated by clonotype found that the dominant clone was not transcriptionally distinct from other non-dominant clones, supporting the observation of a true polyclonal response to immunotherapy.

Differential expression analysis of γ/δ T cells between baseline and follow-up samples for the patient with a complete response demonstrated that these cells became more activated, with increased expression of cytotoxic molecules (*GZMA*, *GZMB*, *GZMM*, *GZMH*), markers of cytotoxic activity (*KLRB1*), and chemokines (*CCL4*, *CCL5*) (**Figure 4E**). Interestingly, these chemokines are also ligands for CCR5, which is expressed more highly on γ/δ T cells from responder patients, hinting at a positive feedback loop for recruitment of these cells into inflamed tissue. γ/δ T cells also expressed less *IL7R* (which typically denotes more immature T cells), less *IL10RA* (which would render the cells resistant to IL10-mediated suppression by Tregs or MDSCs), less *NFKBIA* (demonstrating an increase in NFκB signalling associated with TCR engagement) and less *S100A8*/*S100A9* (which promote accumulation of MDSCs in tumor tissue) (12). Increased expression of *GZMB* and *KLRB1*, and decreased expression of *IL7R* by these γ/δ T cells were all validated at the protein level through mass and flow cytometry measurements (**Figure 4F**).

Overall, these results demonstrate a potential role for γ/δ T cells in mediating the anti-tumor response to PD-1 blockade.

## Discussion

Immune checkpoint blockade has become a standard of care in renal cell and urothelial cancers. However, given that the benefit seen in clinical trials is largely driven by a small subset of patients, there is a need for better biomarkers. This study demonstrates that the circulating immune compartment is a dynamic environment which reacts to exposure to checkpoint blockade. Key limitations include the small sample size, heterogeneity in treatment and primary histology and the fact that while the circulating immune compartment was sampled, we do not have sampling of the tumour microenvironment; therefore, the results presented here should be considered hypothesis-generating. High-dimensional analysis is a suitable tool for biomarker discovery, and even with a small and heterogenous cohort we are observing clear differences between responders and non-responders. In particular, we highlight higher frequencies of naïve CD4+ T cells and lower frequencies of CD161+ Th17 cells and CCR4+ Th2 cells in responders. Similar to the data published by Krieg et al. (3) in melanoma, we find that responders had a lower frequency of effector memory CD4+ T cells.

The frequencies of broad immune populations did not change with exposure to checkpoint inhibitors in our study. Similarly, for patients with melanoma who received checkpoint inhibitors, Pirozyan et al. showed that differences in immune populations are seen between responders and non-responders and are maintained during treatment up to one year after initiation of checkpoint inhibitors (13). However, in our study high-dimensional analysis reveals dynamic changes in the function and frequency of specific subpopulations that occur very early during treatment, before any radiographic changes would be expected. We highlight the role of PD1+ circulating T-cells. We find that PD1+/CTLA4+ T cells have higher Ki67 expression, and that this is significantly increased in non-responders. This is similar to data published by Huang et al. (4), although once the Ki67 expression was normalized for tumour burden the relationship inverted in their study. In addition, the CD4+ T-cells of non-responders had higher expression of TNFα and IFNγ; their PD1+ CD4+ T-cells also expressed lower levels of CCR4. Together with the lower frequency of naïve CD4 T-cells and increased Ki67, this demonstrates a curious pattern of non-responder patients having an overall less naïve and more activated T cell phenotype compared to responder patients. Overall, our study independently validates many findings demonstrated in patients with melanoma and shows that these findings can be found in patients with UC and RCC as well. The role of cytokine production by peripheral immune cells as prognostic biomarkers is an active field of research producing heterogenous results. As research progresses, more nuanced immune signatures such as the ratios between cytokines, or between cytokines and tumor burdens, can yield greater predictive utility and biological interpretation than crude measurement of a single cytokine.

A particularly novel finding in our study highlights the potential role of γ/δ T-cells in an exceptional responder. This patient had a clonal expansion of Vγ9/Vδ2 T-cells following PD-1 blockade. Vγ9/Vδ2 T-cells have been proposed as an effector cell for cancer immunotherapy (14), and there are preclinical studies investigating γ/δ chimeric antigen receptor (CAR) T-cells as potential therapies. In addition, Miao et al. have shown that tumour infiltration by γ/δ-T cells was associated with response in patients with melanoma who received anti-PD1 therapy (15). The role of different γ/δ subsets to both promote tumorigenesis and as potential effector cells in anti-tumour immune mechanism is still poorly understood, and there are significant differences between circulating and tumour infiltrating γ/δ-T cells (16). Nevertheless, the strong clonal expansion of Vγ9/Vδ2 T-cells observed in an exceptional responder to checkpoint inhibition supports further investigation of these cells as potential effectors of response to checkpoint inhibition. Vγ9/Vδ2 is the most frequent TCR variant found in peripheral blood (17). Tosolini et al. (14) showed that Vγ9/Vδ2 T-cells can be found as tumour infiltrating lymphocytes (TILs), and that their frequency did not correlate with that of αβ TILs. Their exact role in the tumour microenvironment remains a topic of active research. In our case, the combination of durvalumab and chemotherapy makes interpretation of the findings more challenging. Ultimately, they should be considered hypothesis-generating and require further validation in a larger cohort.

One limitation of mass cytometry is related to the need to run samples in batches, to allow for normalization of readings and avoid introducing an error from instrument drift over time. In our study, PBMCs were isolated from whole blood and cryopreserved. Our cryopreservation process did not allow for sufficient viability in granulocyte cells for reliable analysis of this aspect of the immune system. The study was also designed to only look at the circulating immune compartment. Although changes in cytokines and cell numbers can lead us to hypothesize on the immune environment of patients who receive checkpoint inhibitors, without serial tissue sampling in tumours and lymph nodes this remains hypothetical.

## Conclusions

Overall, our study supports the use of high-dimensional analysis of PBMCs for biomarker discovery in patients receiving immune checkpoint blockade. Unlike molecularly targeted anticancer therapies that directly affect mutant cells, checkpoint inhibitors have a broad immunomodulatory role. In this setting, looking a broad array of biomarkers allows for both discovery of new potential biomarkers and a better understanding of the underlying immune biology. Despite the small number of patients studied and the heterogeneity between cases, strong signals are observed and warrant further investigation in larger studies. While we can only indirectly infer intratumoral changes based on chemokine expression, our approach allows for serial sampling of the immune system without the morbidity associated with tissue biopsies. High-dimensional analysis of PBMCs may be an ideal platform to generate a broad array of hypotheses and highlight the role of often overlooked subtypes of immune cells.

## Data availability statement

The original contributions presented in the study are included in the article/**Supplementary Material**. Further inquiries can be directed to the corresponding author.

## Ethics statement

The studies involving human participants were reviewed and approved by BC Cancer Research Ethics Board. The patients/participants provided their written informed consent to participate in this study.

## Author contributions

J-ML and MN contributed to the conception and design of the study. J-ML, LN, DK, CK and BE contributed to sample acquisition. PB, EC, MN performed experiments. KC, CS, AW and BE contributed supervision funding and technical expertise. J-ML and MN wrote the first draft; all co-authors provided manuscript revisions and approved the final manuscript. All authors contributed to the article and approved the submitted version.

## Funding

This project was supported by a grant from the Genito-Urinary Medical Oncologists of Canada (GUMOC). J-ML was supported by the University of British Columbia Clinician Investigator Program.

## Acknowledgments

The authors would like to thank Ms. Lejla Gavranovic and Mr. John Mah for their assistance with sample acquisition.

## Conflict of interest 

Author J-ML received honoraria from Bayer, EMD Serono, Pfizer and Tersera. LN received honoraria from Pfizer, Ipsen, Bayer, Merck, Astra Zeneca and research support from Ipsen, Janssen, EMD Serono. KC received honoraria from Astellas, AstraZeneca, Daiichi Sankyo, Janssen, Merck, Novartis, Pfizer, Point Biopharma, Roche, Sanofi, and research support from Astellas, AstraZeneca, Janssen, Merck, Sanofi.

The remaining authors declare that the research was conducted in the absence of any commercial or financial relationships that could be construed as a potential conflict of interest.

## Publisher’s note

All claims expressed in this article are solely those of the authors and do not necessarily represent those of their affiliated organizations, or those of the publisher, the editors and the reviewers. Any product that may be evaluated in this article, or claim that may be made by its manufacturer, is not guaranteed or endorsed by the publisher.
