# Supplementary material for: Comprehensive immune profiling of patients with advanced urothelial or renal cell carcinoma receiving immune checkpoint blockade
Source: Front Oncol. 2022 Sep 13;12:973402. doi: 10.3389/fonc.2022.973402 (PMC9513023; doi:10.3389/fonc.2022.973402)
Supplement: Supplementary file 1 [file Table_1.docx]

Supplementary Table 1: Mass cytometry panel

| ***CyTOF Channel*** | ***Protein Marker*** |  | ***CyTOF Channel*** | ***Protein Marker*** |
| --- | --- | --- | --- | --- |
| 89Y | CD45 |  | 158Gd | CD27 |
| 113Cd | Viability |  | 159Tb | FoxP3 |
| 115In | CD57 |  | 160Gd | CD28 |
| 141Pr | CD196 (CCR6) |  | 161Dy | CTLA-4 |
| 142Nd | CD19 |  | 162Dy | CD66b |
| 143Nd | CD127  (IL-7Ra) |  | 163Dy | CD183 (CXCR3) |
| 144Nd | CD38 |  | 164Dy | CD161 |
| 145Nd | CD138 |  | 165Ho | CD45RO |
| 146Nd | IgD |  | 166Er | CD24 |
| 147Sm | CD11c |  | 167Er | CD197 (CCR7) |
| 148Nd | CD16 |  | 168Er | CD8 |
| 149Sm | CD194 (CCR4) |  | 169Tm | CD25 (IL-2R) |
| 150Nd | CD86 |  | 170Er | Ja18 |
| 151Eu | CD123 (IL-3R) |  | 171Yb | CD20 |
| 152Sm | TCR g/d |  | 172Yb | Ki67 |
| 153Eu | CD185 (CXCR5) |  | 173Yb | HLA-DR |
| 154Sm | CD3 |  | 174Yb | CD4 |
| 155Gd | CD45RA |  | 175Lu | CD14 |
| 156Gd | PD-L1 |  | 176Yb | CD56 |
| 157Gd | GITR |  | 209Bi | CD11b |
